# Autoimmune targeting of key components of RNA interference

**DOI:** 10.1186/ar1959

**Published:** 2006-05-09

**Authors:** Andrew Jakymiw, Keigo Ikeda, Marvin J Fritzler, Westley H Reeves, Minoru Satoh, Edward KL Chan

**Affiliations:** 1 Department of Oral Biology, University of Florida, 1600 S.W. Archer Road, Gainesville, FL, 32610, USA; 2 Department of Biochemistry and Molecular Biology, University of Calgary, 3330 Hospital Drive N.W., Calgary, AB, T2N 4N1, Canada; 3 Division of Rheumatology and Clinical Immunology, Department of Medicine, and Department of Pathology, Immunology, and Laboratory Medicine, University of Florida, 1600 S.W. Archer Road, Gainesville, FL, 32610, USA

## Abstract

RNA interference (RNAi) is an evolutionarily conserved mechanism that is involved in the post-transcriptional silencing of genes. This process elicits the degradation or translational inhibition of mRNAs based on the complementarity with short interfering RNAs (siRNAs) or microRNAs (miRNAs). Recently, differential expression of specific miRNAs and disruption of the miRNA synthetic pathway have been implicated in cancer; however, their role in autoimmune disease remains largely unknown. Here, we report that anti-Su autoantibodies from human patients with rheumatic diseases and in a mouse model of autoimmunity recognize the human Argonaute (Ago) protein, hAgo2, the catalytic core enzyme in the RNAi pathway. More specifically, 91% (20/22) of the human anti-Su sera were shown to immunoprecipitate the full-length recombinant hAgo2 protein. Indirect immunofluorescence studies in HEp-2 cells demonstrated that anti-Su autoantibodies target cytoplasmic foci identified as GW bodies (GWBs) or mammalian P bodies, structures recently linked to RNAi function. Furthermore, anti-Su sera were also capable of immunoprecipitating additional key components of the RNAi pathway, including hAgo1, -3, -4, and Dicer. Together, these results demonstrate an autoimmune response to components of the RNAi pathway which could potentially implicate the involvement of an innate anti-viral response in the pathogenesis of autoantibody production.

## Introduction

The exact mechanisms and causes of autoimmune diseases remain unknown. They are thought to develop when self-reactive lymphocytes escape from tolerance and are activated or when incomplete thymic and/or bone marrow clonal selection or disruption of the anergy of autoreactive lymphocytes perturb the delicate balance of non-self-antigen and self-antigen recognition [1]. The disequilibrium between pro-inflammatory and immunosuppressive cytokines is also thought to contribute to the autoimmune phenomenon [2].

Although our understanding of these specific disease processes is incomplete, human autoantibodies have proven very useful for the discovery, identification, and elucidation of newly described cellular components and macromolecules [3]. For example, the identification and characterization of small nuclear ribonucleoproteins and the spliceosome were made possible through the use of human autoantibodies [4].

Patients with systemic rheumatic diseases commonly produce antibodies against specific classes of highly conserved RNA-protein complexes. These include several known RNA-binding autoantigens, such as SS-A/Ro, SS-B/La, Sm, and U1 RNP [3]. RNA-binding proteins are of interest because they represent a class of novel regulators of gene expression. Their functions include, but are not limited to, transcription, splicing, translation, transport, stability, and degradation.

Recently, human autoantibodies were used to identify and characterize a new protein named GW182 [5]. GW182 is an mRNA-binding protein that is characterized by a highly repetitive glycine (G) and tryptophan (W) domain at the amino terminus. In addition, GW182 is associated with a subcellular structure, the GW body (GWB) or mammalian P body, that is involved in mRNA degradation [6,7]. More recently, knockdown of GW182 and disruption of GWBs were demonstrated to impair RNA interference (RNAi) or RNA silencing [8,9].

RNAi is an evolutionarily conserved mechanism involved in the post-transcriptional regulation of gene expression in many eukaryotes [10]. It was initially recognized as an anti-viral mechanism that protected organisms from RNA viruses [11] or the random integration of transposable elements [10]. However, not until the discovery that plants and animals encode small RNA molecules referred to as microRNAs (miRNAs) did it become apparent that this mechanism was also responsible for the post-transcriptional regulation of gene expression [10,12].

RNAi is triggered by double-stranded RNA (dsRNA) precursors that are rapidly processed into small RNA duplexes of approximately 21 nucleotides in length by a dsRNA-specific endonuclease termed Dicer [10]. These small RNA duplexes commonly referred to as short interfering RNAs (siRNAs) or miRNAs incorporate into the RNA-induced silencing complex (RISC). Upon binding to RISC, one of the RNA strands then disassociates and subsequently activates the complex. The single-strand siRNA/miRNA within RISC then guides and ultimately cleaves or represses the translation of target mRNAs [10].

Some of the proteins most consistently found in RISC are the highly conserved Argonaute (Ago) proteins [12]. There are eight proteins in the human Ago family [13], four of which, hAgo1-4, have been demonstrated to associate with siRNAs/miRNAs in humans [14]. However, only hAgo2 has been demonstrated to possess the catalytic cleavage activity associated with RNAi [15,16]. Interestingly, hAgo2 has been recently demonstrated to associate with GW182 and localize to GWBs [8,9,14,17].

To date, the most commonly identified diagnoses of patients with autoantibodies to GW182 and GWBs are Sjögren's syndrome, mixed motor/sensory neuropathy, and systemic lupus erythematosus (SLE) [18]. However, autoantibodies to GWBs with other antigen specificities have also recently been identified in patient sera [19-22], in particular from a subset of patients with primary biliary cirrhosis [19]. Therefore, the identification of autoantibodies targeting GW182 and GWBs [5,18] and their recent links with RNAi [8,9,14,17] suggest that other components of the RNAi pathway may potentially be targets of autoimmunity. In this report, we show that the previously reported anti-Su autoantibody [23,24] targets hAgo2 and other key components of the RNAi machinery. Furthermore, we demonstrate that anti-Su autoantibodies stain GWBs in human cells. The significance of this study is that it identifies autoimmune responses to components of the RNAi machinery and provides insights into systemic rheumatic diseases associated with the Su antigen.

## Materials and methods

### Antibodies and sera

Human sera used in this study were obtained from serum banks at the Advanced Diagnostics Laboratory, University of Calgary (Calgary, AB, Canada), the University of Florida Center for Autoimmune Diseases (Gainesville, FL, USA), and the University of North Carolina Hospitals (Chapel Hill, NC, USA). Murine sera were obtained from BALB/c mice prior to or after pristane injection [25]. Murine monoclonal antibodies to GW182 (4B6) were from CytoStore Inc. (Calgary, AB, Canada). Human and murine anti-Su autoimmune sera were identified based on specific reactivity to the 100/102- and 200-kDa Su antigens by immunoprecipitation (IP) of radiolabeled K562 (human erythroleukemia) cell extracts, SDS-PAGE, and autoradiography [23]. Prototype human anti-GW182 sera were described previously [5,8,18]. These studies were approved by the institutional review boards and institutional animal care and use committees of the University of Florida, the University of North Carolina, and the University of Calgary.

### Plasmid DNA constructs

The hAgo2 cDNA in pCMV-SPORT vector was obtained from Dr. Tom Hobman (University of Alberta, Edmonton, AB, Canada) [26]. The hAgo1 (clone 30344513; GenBank BC063275) and hAgo4 (clone 4373725; GenBank BF979523) cDNAs in pBluescript were purchased from Open Biosystems (Huntsville, AL, USA). For both hAgo1 and hAgo4, GC-rich regions in the 5'-untranslated region were deleted to enhance the *in vitro *transcription and translation (TnT) reaction. Briefly, the hAgo1 plasmid was digested with EcoRI and SmaI, purified, filled in with Klenow polymerase, and religated. Similarly, the hAgo4 plasmid was digested with NotI and NcoI, purified, and ligated with a linker containing an EcoRV cut site: 5'-GGCCGATATCGTGC-3' and 5'-CATGGCACGATATC-3'. The hAgo3 cDNA (clone CS0DB008YP10; GenBank AL522515) in the pCMV-SPORT6 vector was purchased from Invitrogen (Carlsbad, CA, USA). The Dicer insert (KIAA0928; GenBank X52328) in pBluescript was obtained from the Kazusa DNA Research Institute (Chiba, Japan). The Dicer coding region was recloned by polymerase chain reaction (PCR) amplification using the following two PCR primers: 5'-CGATACAGTCGACGCCACCATGGAAAGCCCTGCTTTGCAACC-3' and 5'-CCAATACGGCACGACAGTC-3'. All constructs were confirmed by DNA sequencing performed by the University of Florida Interdisciplinary Center for Biotechnology Research core laboratory.

### Fluorescence microscopy

Indirect immunofluorescence (IIF) analysis was previously described [8]. Briefly, the primary antibodies to the following proteins were used: GW182 (human serum, 1:200–1:6,000; murine monoclonal 4B6 (neat)); Su (human sera, 1:100–1:500; mouse sera, 1:100–1:500). The secondary antibodies were anti-mouse or human immunoglobulin G fluorochrome-conjugated goat antibodies, Alexa Fluor 488 (1:400) and Alexa Fluor 568 (1:400) (Invitrogen). Anti-Su sera were analyzed initially at 1:100, 1,200, 1:500, and 1:1,000 using HEp-2 cells (Immuno Concepts N.A. Ltd., Sacramento, CA, USA).

### *In vitro *TnT and IP

The protocol for *in vitro *TnT and IP was previously described [18]. TnT reactions were performed in the presence of [^35^S]-methionine. The IPs of hAgo1-4 and Dicer TnT products were performed using 0.5 M NaCl NET/NP40 buffer (50 mM Tris-HCl [pH 7.5], 500 mM NaCl, 2 mM ethylenediaminetetraacetic acid (EDTA), 0.3% Nonidet P-40) and NET2+F buffer (50 mM Tris-HCl [pH 7.4], 150 mM NaCl, 5 mM EDTA, 0.5% Nonidet P-40, 0.5% deoxycholic acid, 0.1% SDS, 0.02% sodium azide) containing Complete protease cocktail inhibitors (Roche, Basel, Switzerland), respectively. Cell labeling and IP of proteins from [^35^S]-methionine-labeled HeLa or K562 cell extracts using human and/or murine sera were described previously [5,23].

## Results and Discussion

### The Su antigen localizes to cytoplasmic structures identified as GWBs

**Figure 1:**
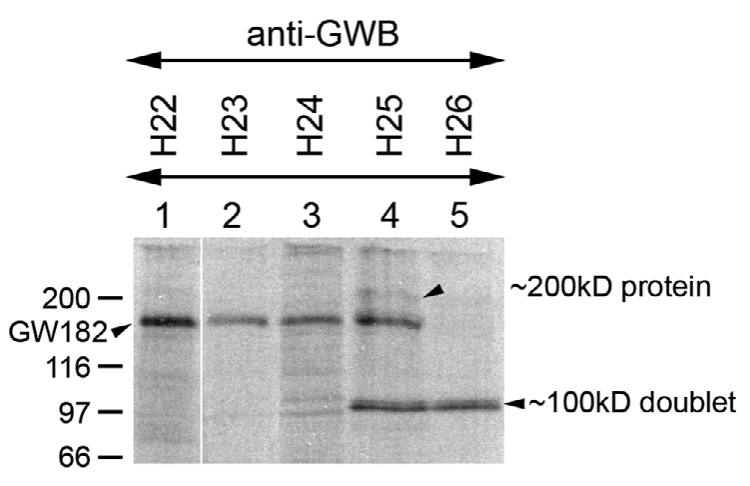
Immunoprecipitation analysis of human GW body (GWB)-positive autoimmune sera. Analysis of human GWB-positive sera demonstrated immunoprecipitation of GW182 from [^35^S]-methionine-labeled HeLa cell extracts (lanes 1–4), except for serum (H26) (lane 5). An approximately 100-kDa protein doublet (lanes 4 and 5) and a weak approximately 200-kDa protein (lane 4) were observed in some of these immunoprecipitations. These immunoprecipitated proteins resembled the immunoprecipitation characteristics of the Su autoantigen. Note that a control normal human serum did not immunoprecipitate any of the proteins described above (not shown).

During our exploration of the biology of GWBs and RNAi, hAgo2, an approximately 100-kDa protein, was demonstrated to coimmunoprecipitate with GW182 and localize to GWBs [8]. Studies using radiolabeled human cell extracts confirmed the presence of an approximately 100-kDa protein that was immunoprecipitated by some human GWB-positive autoimmune sera (Figure 1). Interestingly, we noted that the immunoprecipitated protein exhibited IP characteristics similar to the elusive Su autoantigen, which is part of a macromolecular complex that consists of a 100/102-kDa protein doublet [23]. Based on these findings, we hypothesized that hAgo2 could be the approximately 100-kDa Su autoantigen. If this hypothesis were proven to be true, it would link a known cellular component of the RNAi machinery to autoimmunity and the induction of specific autoantibodies directed toward the Su autoantigen.

Autoantibodies to the Su antigen were initially identified and characterized in 1984 and reported to be specifically associated with SLE in both humans and mice [24,27]. However, a more extensive study later demonstrated that the Su antigen was a frequent target of autoantibodies in a variety of systemic rheumatic diseases [23].

**Figure 2:**
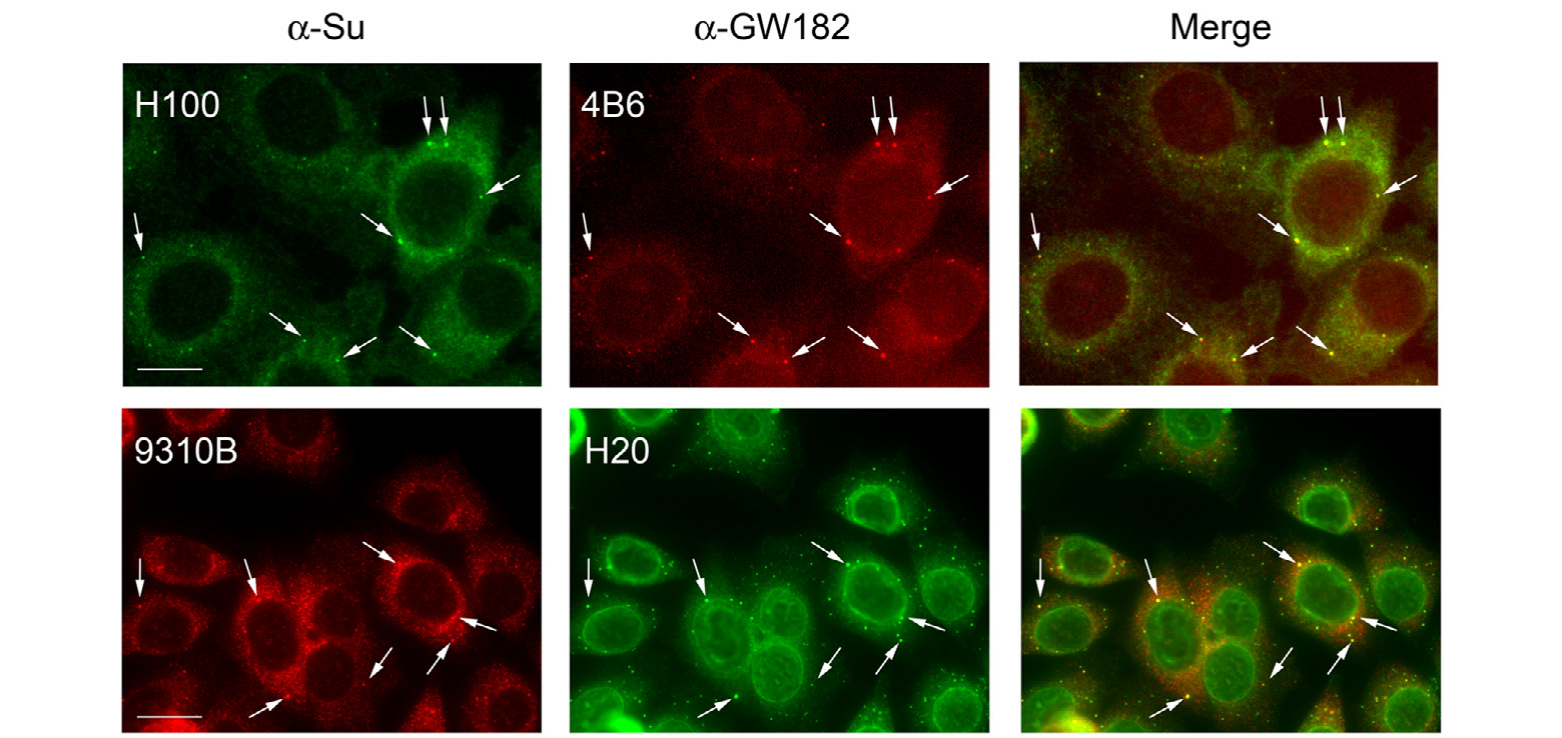
Human and mouse anti-Su sera stain cytoplasmic foci identified as GW bodies (arrows). (top) HEp-2 cells were stained with human anti-Su serum (H100) and anti-GW182 monoclonal antibody (4B6). Bar, 10 μm. (bottom) Hep-2 cells were stained with mouse anti-Su (9310B) and human anti-GW182 (H20) sera. Note that the prototype human anti-GW182 (H20) serum is known to have additional nuclear-envelope antibodies [35]. Bar, 20 μm.

Several monospecific anti-Su human and mouse sera have been demonstrated to exhibit a negative fluorescent anti-nuclear antibody staining, although in a number of cases the sera have been shown to stain the cytoplasm of cells [23]. Knowing that both endogenous and exogenously introduced forms of hAgo2 localized to GWBs [8,9,14,17], we hypothesized that the approximately 100-kDa Su antigen, if it were hAgo2, would also localize to small discrete structures within the cytoplasm of the cells, which are characteristic of GWBs. Initial analysis of a human anti-Su autoimmune serum in HEp-2 cells by IIF demonstrated that the serum stained discrete cytoplasmic foci; these foci were determined to be GWBs by costaining with a GWB marker (Figure 2, top). Because pristane has previously been demonstrated to induce the production of anti-Su antibodies in approximately 50% of BALB/c mice [25], we used an anti-Su serum from a pristane-induced autoimmune mouse as further evidence in support of the potential specificity of anti-Su serum for GWBs. Testing of this serum by IIF independently suggested that the Su antigen is enriched in GWBs (Figure 2, bottom). The failure of a previous report [23] to observe GWB staining was most likely related to the fixation protocol required to preserve GWBs within the cytoplasm. The anti-GWB IIF titers for anti-Su sera ranged from 1:200 to 1:500. In addition to the GWB localization, diffuse cytoplasmic staining was observed for both human and mouse anti-Su sera. This result is not surprising; similar cellular staining patterns have been observed for hAgo2/RISC [14,17], and it has been proposed that hAgo2/RISC shuttles between the cytoplasm and cytoplasmic bodies [17]. Together, these data supported the notion that anti-Su sera were capable of recognizing an antigen that localized to GWBs, a known characteristic of hAgo2.

### The Su antigen is biochemically similar to hAgo2

**Figure 3:**
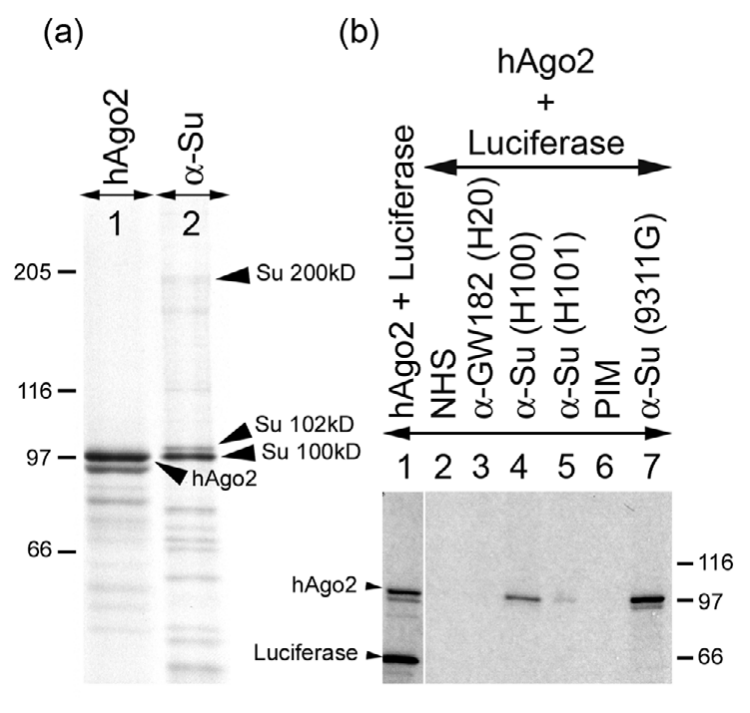
Human and mouse anti-Su sera recognize *in vitro *translated hAgo2 protein. **(a)** K562 cells were radiolabeled and extract was immunoprecipitated with human anti-Su serum (lane 2) and compared with the migration pattern of the *in vitro *synthesized hAgo2 product (lane 1). **(b)** Immunoprecipitation of *in vitro *translated hAgo2 product using human anti-GW182 serum (lane 3), human anti-Su sera (lanes 4 and 5), and mouse anti-Su serum (lane 7) compared with normal human serum (NHS) and pre-immune mouse serum (PIM) (lanes 2 and 6, respectively). Lane 1 represents the *in vitro *transcription and translation mix prior to immunoprecipitation.

In previous biochemical studies, the Su antigen was demonstrated to be a macromolecular complex consisting of 100/102- and 200-kDa proteins [23], the former being consistent with the approximately 100-kDa molecular mass of hAgo2. To confirm that the Su antigen had a molecular mass similar to that of hAgo2, a human prototype anti-Su serum was used to immunoprecipitate the 100-kDa protein from a human (K562) cell extract, after which the immunoprecipitated product was compared with that of an *in vitro *translated hAgo2. SDS-PAGE analysis clearly demonstrated that the Su antigen immunoprecipitated by the human anti-Su serum comigrated with the *in vitro *translated hAgo2 (Figure 3a). Furthermore, human anti-Su sera and pristane-induced autoimmune anti-Su mouse sera immunoprecipitated the *in vitro *translated hAgo2, whereas human and mouse control sera did not (Figure 3b). For this experiment and subsequent IP experiments involving *in vitro *translated products, *in vitro *translated Luciferase protein was added to the IP mix to demonstrate specificity of anti-Su interactions. The inability of the anti-Su sera to immunoprecipitate the *in vitro *translated Luciferase product in the experiment described above demonstrated the specificity of the anti-Su sera for hAgo2. Taken together, these data strongly supported the hypothesis that the approximately 100-kDa Su antigen was hAgo2.

**Figure 4:**
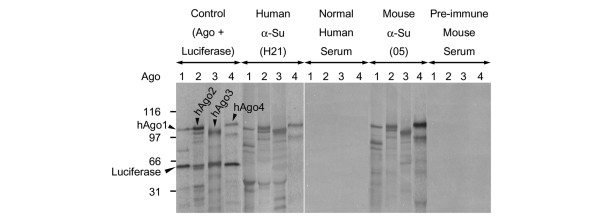
Human and mouse anti-Su sera immunoprecipitate all four Argonaute (Ago) family members. *In vitro *translated hAgo1, -2, -3, and -4 were immunoprecipitated using both human and mouse anti-Su sera in comparison with both normal human and pre-immune mouse sera. As a control, the hAgo *in vitro *transcription and translation mixes were also loaded for migration comparisons.

Human and mouse anti-Su autoimmune sera recognize other members of the Ago protein family. Because the Su antigen has been characterized as a macromolecular complex that consists of a doublet of approximately 100- and 102-kDa proteins [23], it is possible that the 102-kDa protein is a post-translationally modified form of the 100-kDa protein. It has also been suggested that the two components most likely represent distinct polypeptides, because a few anti-Su sera have been identified that recognize the 100-kDa, but not the 102-kDa, component [23]. This observation prompted us to test whether other members of the Ago protein family were targeted by anti-Su sera. IP of full length *in vitro *translated hAgo1, -3, and -4 using either human anti-Su autoimmune serum or pristane-induced autoimmune mouse serum demonstrated that either sera was capable of immunoprecipitating these additional Ago family members in addition to hAgo2 (Figure 4). The inability of the anti-Su sera to immunoprecipitate the *in vitro *translated Luciferase product once again demonstrated the specificity of the binding of anti-Su sera for the family of Ago proteins in the IP reaction. Analysis of five additional human anti-Su (hAgo2-positive) sera similarly demonstrated that each serum was capable of recognizing the other three Ago family members. These results were not surprising and are consistent with the concept of inter-molecular epitope spreading [28], because Ago family members are known to reside and associate together in a macromolecular RNA silencing complex [14,17]. It is also possible that the sera react with a common epitope in the Ago proteins by virtue of the high degree of sequence homology (approximately 80% identity) exhibited between the Ago proteins [13]. Regardless of the type of interaction that occurs between the autoimmune sera and these additional Ago family members, the important point to note is that the anti-Su autoimmune sera target key components of the RNAi machinery.

### Human anti-Su autoimmune sera recognize Dicer

**Figure 5:**
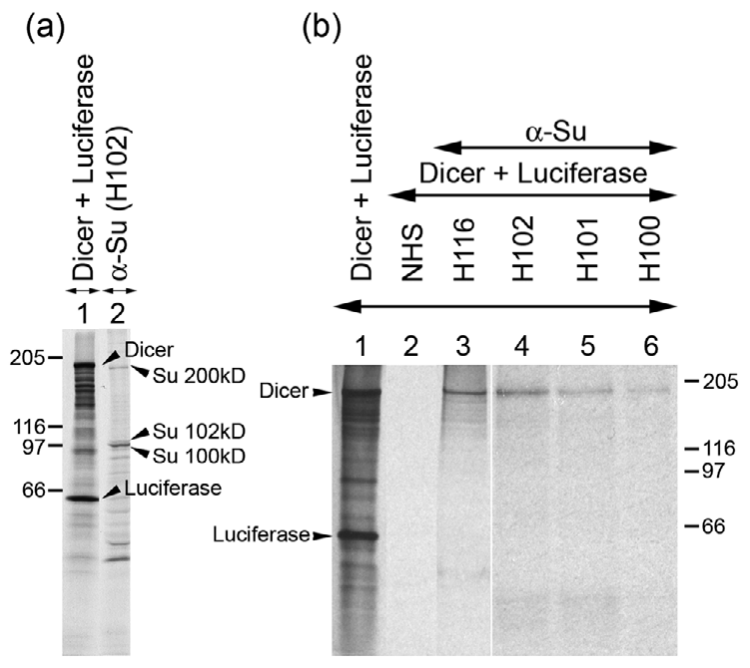
Human anti-Su sera immunoprecipitate *in vitro *translated Dicer protein. **(a)** K562 cells were radiolabeled and extract was immunoprecipitated with human anti-Su serum (lane 2) and compared with the migration pattern of the *in vitro *synthesized Dicer product (lane 1). **(b)** Immunoprecipitation of *in vitro *translated Dicer product using human anti-Su sera (lanes 3–6) compared with normal human serum (NHS) (lane 2). Lane 1 represents the *in vitro *transcription and translation mix prior to immunoprecipitation.

Besides the core Ago proteins and the small RNA components of RISC, other proteins have been identified in higher molecular mass forms of the purified complex [10]. These larger forms are thought to be due to the weak and/or transient association of proteins involved in the initial processing of dsRNA (for example, Dicer) [10]. The sedimentation behavior of the Su antigen suggests that it exists as a large protein complex that carries 100/102- and 200-kDa proteins [23]. With the identification of the approximately 100-kDa protein as hAgo2, the obvious candidate for the 200-kDa protein was Dicer. Dicer, an approximately 210-kDa protein, has been previously demonstrated to associate with hAgo2 [26]. To test whether the 200-kDa component of the Su antigen had a molecular mass similar to that of Dicer, a human anti-Su serum was used to immunoprecipitate the 200-kDa protein from a human (K562) cell extract, after which the immunoprecipitated product was compared with that of an *in vitro *translated Dicer. SDS-PAGE analysis demonstrated that the 200-kDa Su antigen immunoprecipitated by the human anti-Su serum comigrated with the *in vitro *translated Dicer (Figure 5a). Furthermore, the specificity of the reaction was demonstrated when the human anti-Su sera immunoprecipitated the *in vitro *translated Dicer, but the human control serum did not (Figure 5b). The inability of the anti-Su sera to immunoprecipitate the *in vitro *translated Luciferase product also demonstrated the specificity of the anti-Su sera for Dicer. Cumulatively, these data strongly suggested Dicer as an additional molecular target of anti-Su sera; however, further studies, such as analysis of the 200-kDa protein by IP-mass spectroscopy, will be needed to determine whether Dicer is the true 200-kDa Su antigen.

### Serological characteristics of anti-Su autoimmune sera

To ascertain a broader perspective of the serological properties of anti-Su sera, 22 human and seven pristane-induced mouse anti-Su sera were further characterized and compared with other human Su-negative autoimmune (*n *= 10) or normal human (*n *= 10) and mouse (*n *= 6) sera (Table 1). Autoimmune anti-Su sera were confirmed by IP of the characteristic macromolecular complex, which consists of the doublet of approximately 100/102- and 200-kDa proteins. IIF staining in HEp-2 cells demonstrated that of the 22 human anti-Su sera, 20 (91%) stained cytoplasmic foci that were characteristic of GWBs. Random selection of seven of these positive sera demonstrated that all seven (100%) showed colocalization with monoclonal antibodies to GW182. Of the human anti-Su sera, 91% (20/22) and 36% (8/22) recognized *in vitro *translated hAgo2 and Dicer, respectively. Similarly, 100% (7/7) of the seven pristane-induced autoimmune mouse sera also stained GWBs, and of the tested sera, 100% (5/5) were hAgo2-positive; however, none (0/2) were Dicer-positive. None (0/16) of the normal human and pre-immune mouse sera stained cytoplasmic structures characteristic of GWBs, and none (0/10) of the Su-negative autoimmune sera stained GWB structures. Of the tested sera, none were reactive with *in vitro *translated hAgo2 (0/24) or Dicer (0/9). The significance of these results is that anti-Su autoimmune sera appear to be uniquely defined by their ability to stain GWBs within the cytoplasm of cells and recognize the hAgo2 protein, thus making them readily identifiable. During the course of our serological studies, we also determined that one of the two prototype human anti-GW182 sera was also positive for anti-Su antibodies, further demonstrating the close association and potential overlap between these two sets of autoimmune sera.

In summary, these data identify the Su autoantigen as a macromolecular complex closely associated with the GWB structure and the RNAi pathway. Furthermore, these data demonstrate that anti-Su autoantibodies target key components of the RNA silencing machinery, in particular hAgo2. However, further studies will be required to better ascertain the extent of anti-Su autoantibody reactivity with other members of the Ago family and definitively determine whether Dicer is unambiguously the 200-kDa protein commonly associated with the Su antigen. Regardless, the association between RNAi and systemic autoimmune diseases is potentially significant due to the growing connection between RNAi and viruses. RNAi is an innate anti-viral response found in certain plant and invertebrate species and remains an evolutionarily conserved mechanism in many eukaryotes. Its role as a natural anti-viral response in mammals has been postulated and is supported by the evidence that mammalian viruses encode suppressors of RNAi [29,30]. Even though at present the clinical significance of anti-Su autoantibodies is not apparent, it is intriguing to speculate that viruses may promote the development of autoimmunity via their association with components of the RNAi pathway. Given that RNAi is linked to the interferon system [31], viral suppressors inhibit RNAi [29,30], viruses encode miRNAs [32], and virus-like particles associate with GWB/P-body components [33], it is not surprising that GWBs and thus RNAi protein and/or nucleic acid components develop into targets of autoimmunity.

## Conclusion

Our work links key components of the RNAi machinery with autoimmune disease. Moreover, our study identifies a murine model of autoimmunity that has the potential to advance our understanding of autoimmune responses to components of the RNAi pathway. Interestingly, pristane treatment of mice has been demonstrated to activate endogenous retroviruses [34]. Therefore, future studies with a focus on the interplay among RNAi, viruses, and autoimmunity should help clarify whether RNAi itself or its association with an invading virus is directly linked to the pathogenesis of systemic autoimmune diseases.

## Abbreviations

Ago = Argonaute; dsRNA = double-stranded RNA; EDTA = ethylenediaminetetraacetic acid; G = glycine; GWB = GW body; IIF = indirect immunofluorescence; IP = immunoprecipitation; miRNA = microRNA; PCR = polymerase chain reaction; RISC = RNA-induced silencing complex; RNAi = RNA interference; siRNA = short interfering RNA; SLE = systemic lupus deerythematosus; TnT = transcription and translation; W = tryptophan.

## Competing interests

The authors declare that they have no competing interests.

## Authors' contributions

AJ carried out the initial IIF and TnT-IP studies, participated in the serological testing and in the design of the study, and drafted the manuscript. KI carried out TnT-IP studies, completed the serological testing, and participated in the design of the study. MS carried out the IP studies and participated in the design of the study. MJF, WHR, and MS collected and classified the sera and also helped edit the manuscript. EKLC conceived of the study, participated in its design and coordination, and edited the manuscript. All authors read and approved the final manuscript.

## Figures and Tables

**Table 1:** Serological features of human and mouse anti-Su sera

	IIF	K562 cell extract IP		TnT IP	
Sera	GWB foci	100/102-kDa Su	200-kDa Su	hAgo2	Dicer

Human anti-Su (*n *= 22)	91%	100%	82%	91%	36%
Mouse anti-Su (*n *= 7)	100%	100%	100%	100%^a^	0%^b^
Human anti-GW182 (*n *= 2)	100%	50%	50%	50%	100%
Human autoimmune non-anti-Su (*n *= 10)	0%	0%	0%	0%	0%^c^
Normal human (*n *= 10)	0%	0%	0%	0%	0%^c^
Pre-immune mouse (*n *= 6)	0%	0%	0%	0%^c^	0%^d^

^a^*n *= 5, ^b^*n *= 2, ^c^*n *= 4, ^d^*n *= 1. GWB, GW body; IIF, indirect immunofluorescence of HEp-2 cells; IP, immunoprecipitation; TnT, *in vitro *transcription and translation.
